# Quality of Online Information Regarding Cervical Cancer

**DOI:** 10.7759/cureus.9511

**Published:** 2020-08-01

**Authors:** Jessica Q Dawson, Janine M Davies, Paris-Ann Ingledew

**Affiliations:** 1 Family Medicine, Faculty of Medicine, University of British Columbia, Vancouver, CAN; 2 Medical Oncology, British Columbia Cancer Agency, Vancouver, CAN; 3 Faculty of Medicine, University of British Columbia, Vancouver, CAN; 4 Radiation Oncology, British Columbia Cancer Agency, Vancouver, CAN; 5 Surgery, Faculty of Medicine, University of British Columbia, Vancouver, CAN

**Keywords:** cervical cancer, patient education, internet, online health information, information quality

## Abstract

Introduction

The internet is an important source of health information, and yet the quality of the resources that patients’ access can vary widely. Previous research has evaluated the quality of information for several types of cancer; however, this has not yet been done for cervical cancer beyond treatment information. The goal of this project was to systematically evaluate the quality of resources for cervical cancer information available against a range of metrics, including content breadth and accuracy, readability, and accountability.

Methods

An internet search was performed using the term “cervical cancer” using Google and two meta-search engines, Dogpile and Yippy. The top-100 websites returned across all three engines were evaluated using a validated structured rating tool.

Results

Only 32% of websites disclosed their author and only 38% used citations, while 64% of websites had been updated in the last two years. Readability was at university-level or higher for 19% of websites, and high-school level for 78%. Coverage was highest for etiology and risk factors (93% of websites) and prevention strategies such as pap smears and vaccines (92%); coverage was lowest for prognosis (49%), staging (52%), side effects (47%), and follow-up (25%). When a topic was covered the information was predominantly accurate, and few websites had inaccurate information. At least one social-media platform was linked to by 79% of websites.

Conclusions

This project highlights the strengths and limitations in the quality of the top-100 informational cervical cancer websites. These findings can inform the dialogue between health care providers and patients around selecting and evaluating information resources. These findings can also inform specific improvements to make online resources for cervical cancer more accessible, comprehensive, and relevant to patients.

## Introduction

Information-seeking and decision-making related to cervical cancer may occur throughout the life of a woman or a person with a cervix. Cervical cancer is the 13th most commonly diagnosed cancer in Canada [1]. Screening programs begin at age 25 and are estimated to have greater than 60% participation, and human papillomavirus (HPV) vaccination is now recommended for girls and boys as young as nine [1]. In Canada, roughly half of cervical cancers are diagnosed in patients under 50, although older age is strongly associated with an increase in the severity of disease at the time of diagnosis [1]. Educational resources on cervical cancer are therefore of importance to patients across age groups, with the general population seeking information regarding prevention, immunization, and screening, and for those who have received or are supporting someone with a diagnosis of cervical cancer seeking information on staging, treatment, and prognosis. 

Patients are increasingly likely to utilize online information and social-media resources to access information related to their health. One study found that upwards of 9% of cancer-related keyword searches are related to gynecological cancers; of those, one third were related to cervical cancer, making it the second most common gynecologic cancer search [2]. Online health information seeking is common among younger patient groups, and is gradually increasing among older patients as well [3,4]. Patients are also turning to social media for information about specific conditions, or to form communities with others who share their experiences [5,6]. 

A significant amount of online medical information is not peer-reviewed and may contain inaccurate or misleading information [7]. This has serious implications for decision making, as a large proportion of patients, including those with gynecological cancers, report that the information they find online influences their treatment decisions [8]. Despite the increasing use of the internet for health information, many patients report difficulties finding or interpreting health information online [9]. The perceived quality of online health information depends not only on the information’s accuracy and trustworthiness, but also features associated with its presentation, such as website interactivity and usability [10].

Little work has been done to evaluate the quality of information about cervical cancer available through websites or social media. One study of the credibility, accuracy and ease of use of forty-six websites presenting information on cervical cancer treatment options found a large variation in quality, and the authors expressed concerns about the suitability of the content for lay patient readers [11]. Another study found the online information for cervical cancer chemotherapy options to be largely superficial and of limited educational use [12]. Both of these studies focused on treatment option information; however, cervical cancer patients have diverse informational needs that evolve throughout the course of the disease and treatment [13,14]. More work needs to evaluate the quality of information across the breadth of topics that patients’ access, including etiology, prevention, diagnosis, and prognosis. 

This paper presents a systematic evaluation of the quality of cervical cancer information resources available online against a range of metrics, including content breadth and accuracy, readability, and accountability using a validated tool. To explore the possible expansion of this tool to social media content, this work also examined the extent to which the organizations behind the websites examined in the study also maintained a social media presence to engage with patients.

## Materials and methods

An internet search was conducted using the term “cervical cancer” in Google and two meta-search engines (Yippy and Dogpile) on April 25, 2018. These engines were chosen for consistency with previous studies from our research group [15-17]. Briefly, Google is used due to its popularity in English speaking countries, while Yippy and Dogpile compile results across an aggregate of engines (including Google, Bing and Yahoo). This accounts for a range of possible search results that a user might encounter when using the engine of their choice, while also weighting Google more heavily to account for its overall popularity [15]. The search was conducted in the Chrome browser. To minimize any personalization of search results, the cache was cleared and private browsing was enabled to block the influence of location. All of the URLs returned from each search were recorded to a maximum of 500, and were assessed for inclusion to the study. Each website was included only once (by recording the top-level domain), even if more than one web page from that site appeared in the results. Websites containing content specifically intended to provide patients with information about cervical cancer that were freely available without a subscription were included. All duplicates, blogs, primary news, academic articles, and websites with only physician-targeted or healthcare-provider targeted content, were excluded. As were websites that appeared to contain no information relevant to cervical cancer. Social-media content and non-text media (such as podcasts or videos), which were outside the scope of the current evaluation tool, were also excluded.

An average rank order for each website was calculated across all three engines. The first 100 websites were then compiled as the “top-100” websites, representing the websites a patient would be most likely to encounter.

The top-100 websites were evaluated using a structured rating tool designed to assess the accountability, site interactivity and organization, reading level, content coverage and content accuracy of informational, text-based websites. The tool was developed in 2009 through a detailed review of available resources for evaluating the quality of medical information on the internet and adaptation of several existing guidelines and validated tools [18]. It has been previously validated for inter-rater reliability and usability by our research group, and has been used to evaluate online information quality for other cancers [15-17]. Accountability criteria are derived from the Health on the Internet (HON) code principles and the DISCERN scale, an instrument designed to assist those without content expertise in evaluating written health information [19,20]. Interactivity and site organization criteria are adapted from the Abbot’s scale [21]. Readability was measured using the Flesch-Kincaid (FK) grade level and the SMOG index [22,23]. FK and SMOG scores are calculated using direct text input on Read-able.com; for consistency, text from the definition, diagnosis and treatment sections (if present) of each website were used for scoring. The tool, including the details of its metrics and scoring, has been described in detail in a prior publication [16].

Content coverage and accuracy were assessed for 11 categories: definition, incidence and prevalence, etiology and risk factors, symptoms, prevention, detection and workup, treatment, prognosis, stage, treatment side-effects, and follow-up. To select these categories and develop a scale for rating the accuracy of the content, information was reviewed and summarized from the Canadian Cancer Society (www.cancer.ca), UpToDate (www.uptodate.com), and the National Institutes of Health (www.cancer.gov). A consensus document was created through iterative review and discussion by the authors, which include two oncologists. A summary of the essential content and detail necessary for the complete accuracy of each topic is summarized in Table 1. A website was judged to have covered a topic provided any information on that topic was identifiable. A website was described as completely accurate when the topic included all the necessary components and was in complete agreement with the above sources, mostly accurate if minor components were missing or there were minor inaccuracies, and mostly inaccurate if the information was not present or not in agreement.

**Table 1:** Minimum information required for each content category to be considered completely accurate

Topic	Required information
Definition	Cancerous or abnormal or malignant cells, AND anatomical location of origin
Incidence or Prevalence	Statistics accurate within 5 years: Approximately 13,000 new cases per year in the United States, OR 0.6% lifetime risk, OR equivalent numbers for website country of origin, as referenced by national cancer statistic reporting agencies
Etiology or Risk Factors	HPV infection as major risk factor, AND at least one additional risk factor that either: a) increases risk in with HPV (high parity, tobacco exposure, long-term oral contraceptives, OR b) increases risk of HPV infection (immune suppression, sexual activity at a young age or greater life time number of sexual partners)
Prevention	Method to protect against HPV infection (e.g. vaccination), AND regular screening at appropriate ages
Symptoms	Must include abnormal vaginal bleeding or discharge, AND pelvic pain or pain during sexual intercourse
Detection or workup	Tissue diagnosis by biopsy; may include discussion of screening tests leading to colposcopy and biopsy
Staging	Staging is from 0 or I – IV based on lesion size, depth and extent of spread (stage-specific definitions not required)
Treatment	Dependent on staging and clinical picture, AND treatments may or may not include surgery, radiation, or chemotherapy, AND minimal description of treatment and when used (e.g. early vs. later stage)
Prognosis	Dependent on stage, AND a survival statistic overall or by stage, as referenced by national cancer statistic reporting agencies
Treatment Side-effects	At least one specific side effect discussed
Follow up	Types of follow up that may be required given treatment type, OR reasonable post-treatment screening schedule

In addition to the metrics evaluated by the existing tool, we also recorded the number of social media platforms to which each of the websites linked and maintained a presence, and the number of websites hosting YouTube videos targeted to cancer patients. 

To determine the inter-rater reliability of website evaluation, two reviewers independently rated a random sample of 10 websites. The inter-rater reliability for each item of the tool was found to be > 0.8, and disagreements were discussed with the co-author and resolved by consensus. Given the high level of initial agreement, following this check one of the reviewers then independently applied the tool to the remaining 90 websites. The results were analyzed through descriptive statistics.

Institutional approval for this project was obtained from the UBC Behavioural Research Ethics Board (H18-00865).

## Results

**Figure 1:**
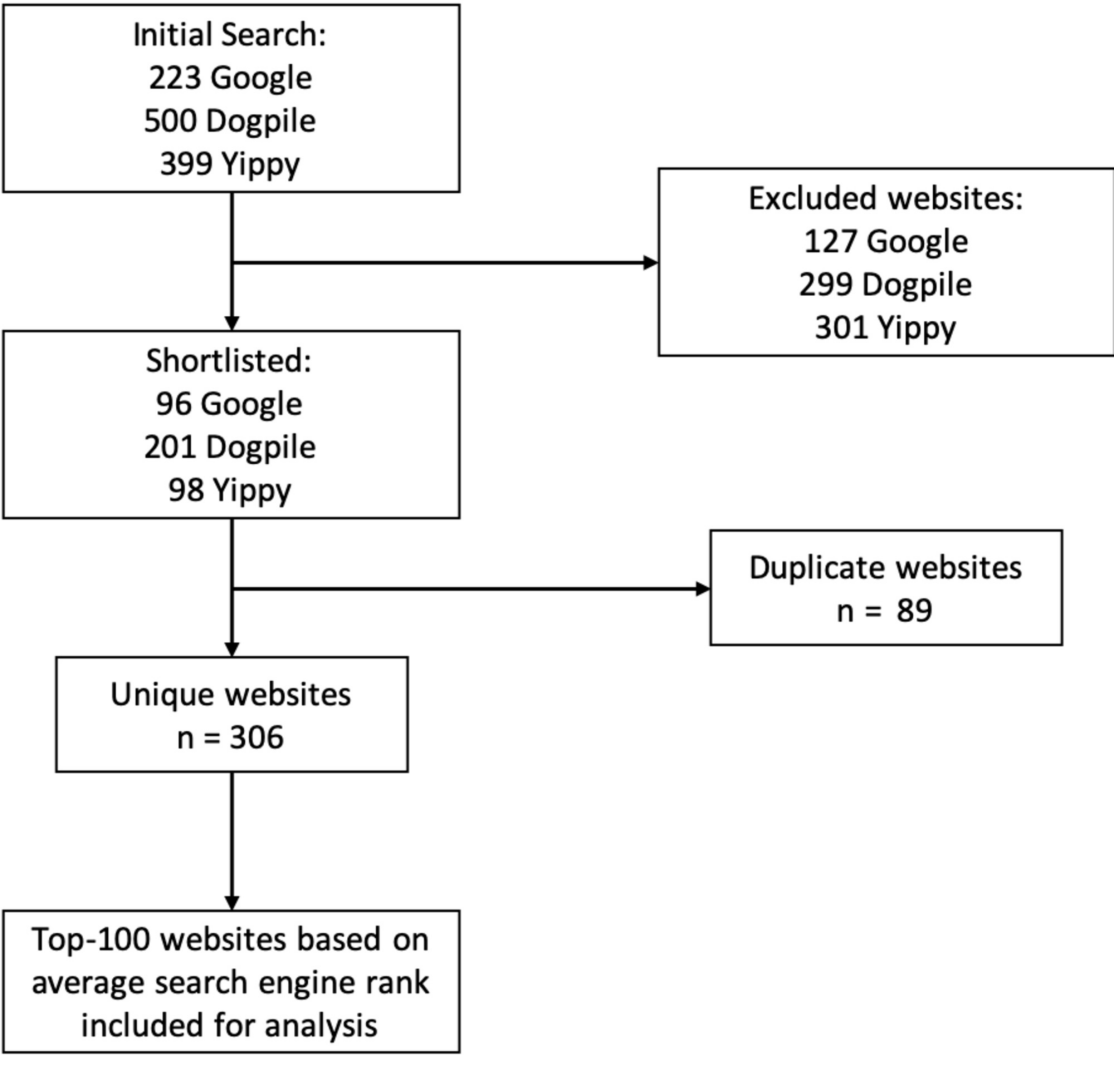
Flow diagram showing inclusion and exclusion of websites for analysis

The search term for “cervical cancer” returned 223 recordable results from Google and 399 from Yippy; recording of URLs from Dogpile was stopped at 500. After applying the exclusion criteria, 96 results remained from Google, 98 from Yippy, and 201 from Dogpile, resulting in a total of 306 unique websites once duplicates were removed. The average rank across the three engines was then used to compile the top-100 websites. A flow diagram summarizing the inclusion and exclusion of websites is shown in Figure 1.

Affiliations and disclosures

Website affiliation was evaluated in terms of primary affiliation and disclosure of ownership. Primary affiliation was most commonly commercial (42% of websites), followed by non-profit or charitable organizations (32%), government organizations (19%) and academic centers or research hospitals (7%). Disclosures of ownership, sponsorship and/or advertising were available on 87% of websites.

Accountability

Accountability was evaluated across four areas: disclosure of authorship, use of citations, links to external information, and information currency. Authorship was disclosed in 32% websites. Of the websites providing authorship, 72% also gave the author’s credentials and 66% gave the affiliation. Citations for the information presented were provided by 38% of websites: of those, 82% had two or more reliable sources, 13% had only one reliable source, and 5% had no reliable sources among their citations. Sources classified as reliable included peer-reviewed publications or peer-reviewed websites like UpToDate, academic or government sites, textbooks or similar resources. A date of creation or date of last modification (or both) was included on 88% of websites: of those, 73% were current within the previous two years, 14% between two and four years ago, and 14% more than four years ago. External links to other sources of information (not including advertisements) were included on 63% of websites, and more than 50% of these links functioned on all but two websites.

Interactivity

Interactivity was assessed in terms of common interactive tools that support patient learning and exploration of information. Internal search engines were widely available (91% of websites), but other interactive features were less common: 35% provided audio/video support, 33% provided support via a webform or email contact for patient questions, 23% provided discussion boards, and 21% provided interactive educational tools (such as cancer knowledge quizzes or symptom-based exploration).

Site organization

Site organization was evaluated through five common design features known to make information more accessible and navigable for users. Headings, subheadings and internal hyperlinks were used by nearly every website (100%, 99%, and 98% of websites, respectively). Pictures or diagrams to supplement written information were provided by 64% of websites. More than half (65%) were free of ads. Commercially affiliated websites were the most likely to have ads (29 of 42); only five (of 26) non-profit websites and one (of six) academic or university-affiliated websites had ads, and all of the government websites were ad-free. 

Readability

The information on most websites was presented at a high-school reading level or higher. On the Flesch Kincaid (FK) score, 19% of websites were at a university level, 78% were at a high school levels (8^th^-12^th^ grade), and only 3% were at an elementary school level (7^th ^grade or less). On the SMOG index, 5% of websites were at a university level, 90% at a high-school level, and 5% at an elementary school level. 

Content coverage and accuracy

**Figure 2:**
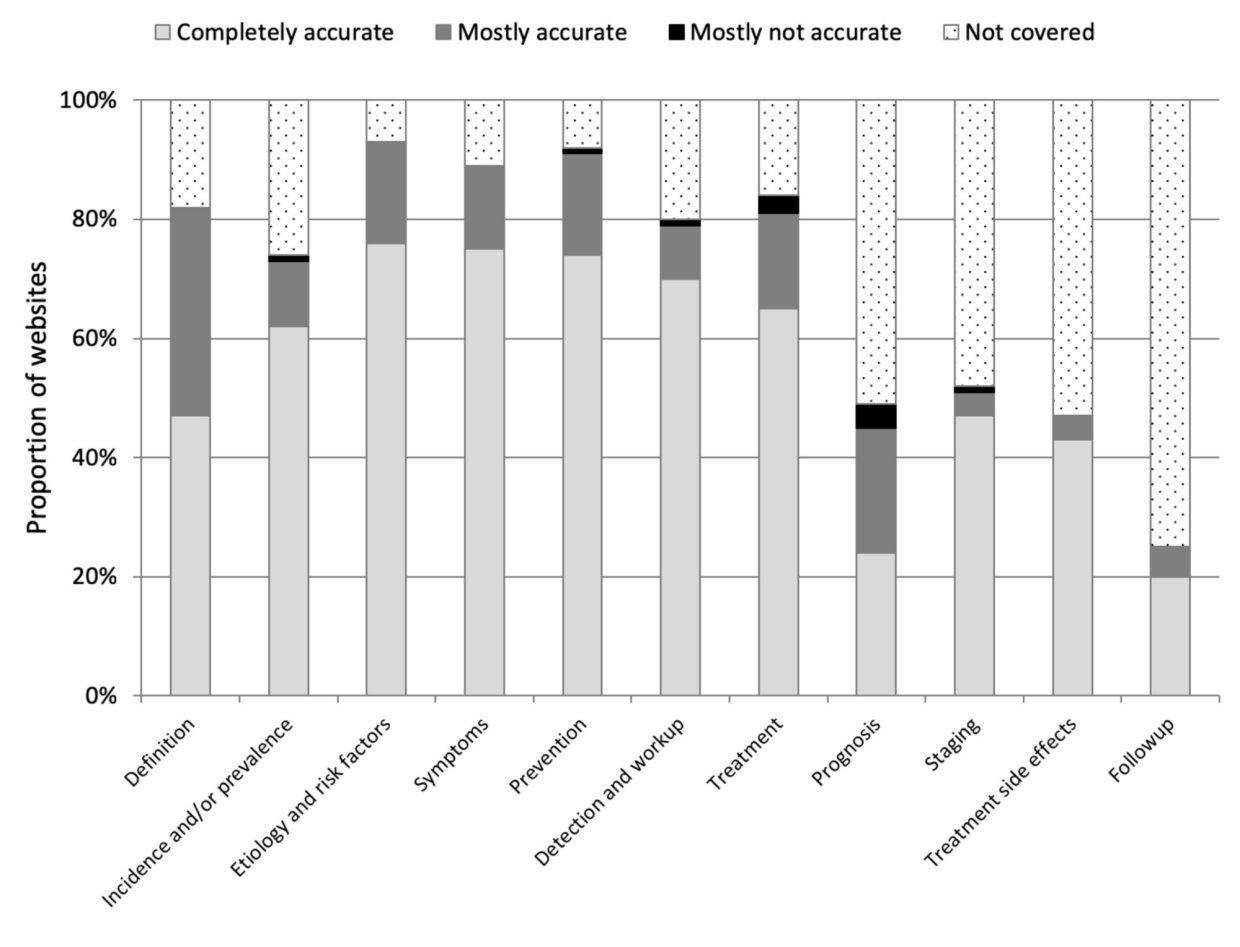
Content categories covered by each website, and the accuracy of the information for each topic covered

Content coverage and accuracy were assessed for 11 categories of information about cervical cancer shown in Figure 2. Websites provided the most coverage for etiology and risk factors (93% of websites) and prevention strategies such as pap smears and vaccines (92%). Coverage was notably lower for staging (52%), prognosis (49%), treatment side effects (47%), and follow up (25%).

Only six websites covered every topic with complete accuracy. The majority of websites had completely accurate information, and nearly all the remainder had mostly accurate information. Notable exceptions were the definition of cervical cancer (completely accurate in only 57%), and prognosis (completely accurate in only 49%). A score of mostly accurate was usually due to correct but incomplete information. For example, many websites that covered prognosis discussed the high survival in early-stage cervical cancers, but then neglected to include discussion of late-stage cervical cancers. Only a small number of websites contained information we judged to be mostly inaccurate for one or more topics.

A score was also assigned for the overall objectivity of the content provided. The majority of websites (87%) were found to be free of any significant bias or opinion. 

Overall quality

An overall score for each website was calculated by combining measures for affiliation, disclosure, accountability, interactivity, site organization, content coverage and accuracy metrics (readability was excluded). The maximum possible score was 60. Scores ranged from 57 (www.cancer.org) to 9 (http://www.oasisofhope.com). The first 10 websites by search engine rank in the top-100 and their associated quality score is shown in Table 2. The average score across all evaluated websites was 36.6 (median 36.0).

**Table 2:** The first ten cervical cancer websites by search-engine average rank in the top-100, with associated quality scores

Average search-engine rank in top-100	Website URL	Quality score (max 60)
1	https://www.webmd.com	52
2	https://en.wikipedia.org/wiki/Cervical_cancer	51
3	https://www.cancer.gov	50
4	https://www.cancer.org	57
5	https://www.cancer.net	53
6	https://www.medicinenet.com	54
7	https://www.emedicinehealth.com	52
8	http://www.cancerresearchuk.org	55
9	https://www.healthline.com	48
10	https://www.womenshealth.gov	30

Seven of the top-10 websites by score also had an average rank within the first 10 search results and appeared in the table above. However, the remaining three top-10 scoring websites (https://www.cancercouncil.com.au, http://www.cancer.ca and https://www.medicalnewstoday.com), with quality scores of 56, 52 and 51, respectively, have average ranked positions of only 64, 14 and 50.

Social media linkage

Linkages to social media were assessed: 79% of websites linked to at least one social media platform on which the organization maintained a presence, with an average of 4.1 platforms per website. Facebook (77% of websites) and twitter (75%) were the most common social media platforms used by the websites; other platforms included YouTube (50%), Instagram (40%), Pinterest (30%), LinkedIn (27%), Google+ (23%), Flickr (4%) and Snapchat (2%). 

Of the websites hosting videos on YouTube, 66% focused on hosting videos with information on cancer or other health-related topics directed at patients or general public, 10% focused on videos directed at physicians or health professionals, and 10% had both. The remainder contained only videos on topics not directly related to providing health information.

## Discussion

The goal of this study was to evaluate the quality of online resources for cervical cancer. A validated, structured rating tool was applied to 100 websites to assess accountability, currency, interactivity and organization, content coverage and accuracy, and readability. The results demonstrate a large variation in quality between websites.

Accountability was assessed by disclosure of authorship, use of citations, links to external information, and information currency. A date of creation or last modification was provided on most websites, but over a quarter of websites were two or more years old. Authorship was disclosed on only a third of websites. Similarly, less than half of the websites provided citations for the information presented. External links to other sources of information were a more common feature and predominantly current. These results show minimal to moderate gains compared to Selman et al.’s 2006 study of online cervical cancer resources, in which 67% of the websites had a date of modification or creation and 30% referenced information [11]. Studies of online resources of other cancers have noted similar deficiencies in authorship disclosure, use of references, and information currency [24,25]. Such accountability markers are important because patient’s perception of the authority of a website’s author, and to a lesser extent, the use of references and external links, and the currency of the information, have all been found to influence the patient’s trust in online health information [10]. Irregularly updated content also puts patients at risk of relying on obsolete information in their decision-making. 

Most websites in this study employed basic design features such as headings, subheadings, internal hyperlinks, and internal search, although other features such as diagrams and discussion forums were less common. Non-profit, government, and academic or university websites tended to be ad-free, as were a small proportion of the commercial websites. Such design features - particularly clear layouts, ease of use, and interactivity - have been found to have positive effects on trust or credibility, whereas advertising has a negative effect [10]. Patients have also been reported to discount high-quality information because of poor website design [26]. These are, therefore, important considerations for organizations seeking to disseminate high-quality information and gain patient trust. 

Most websites presented information at a reading level well beyond what the average Canadian can understand. An elementary school reading level no higher than grade five or six, is widely recognized as the most appropriate for health information [27]. In our study, only three websites had an elementary FK grade level, with the majority presenting information at a high-school grade level or higher. A certain level of technical language is often required to accurately describe complex health topics, but this can make online health information significantly less accessible to patients. Medical educators should be cognisant of reading levels and employ online reading level calculators to check the reading level when developing patient resources. In addition, supportive strategies like glossaries and visual aids can also be employed to further improve the accessibility of the information.

Most topics likely to be of interest to women or people with a cervix at any stage of life were well covered, including cervical cancer etiology, risk factors, symptoms and prevention. This is not surprising, given the public health support for and relatively high patient participation in cervical cancer prevention and routine screening programs in many Western countries [28]. However, the coverage of topics more relevant to patients with cancer was mixed. Noh et al. found that at the time of diagnosis and treatment, cervical cancer patients most often sought information about diagnosis, stage and prognosis, side effects, and etiology and prevention; after diagnosis participants also became interested in self-care and follow up medical tests [13]. Similarly, Okuhara et al. found that users looking for information about cervical cancer were most interested in prognostic information [14]. Our study found good coverage of treatment and diagnosis, but poor coverage of staging, prognosis, side effects, and follow up. When a topic was covered, the information was predominantly accurate and consistent with our reference sources. The most common cause for a reduced accuracy score was incomplete information, and this was most often for the definition or prognosis of cervical cancer. These results are in line with two previous studies of cervical cancer websites: Markman found that markedly erroneous information was present in less than 5% of sites, and Selman et al. also identified missing information as the main reason for website inaccuracy [11,12]. Superficial depth and incomplete information may, therefore, be a persistent limitation of many online cervical cancer resources. Other work has found that many patients are dissatisfied with the information they receive from their physicians, or feel insufficiently informed to fully participate in shared decision-making [29,30]. Altogether, these findings suggest that a general search on cervical cancer may elicit results that are too narrow or superficial to fully meet every patients’ informational needs. Thus, patients may require more deliberate guidance from their healthcare providers towards appropriate resources, and education on how to search for resources that address their specific circumstances. 

Finally, the websites with the highest quality scores were not always the highest ranked by search algorithms. Seven of the top-10 websites by score also had an average rank within the first 10 search results, meaning that these would be likely to appear on the first page of search results. However, the remaining three top-10 scoring websites appeared considerably further down in the search engine results, and therefore may be less likely to be seen. While these results demonstrate some congruence between quality and search engine rank, this finding also suggests that patients and their health care providers cannot rely on search engines alone to direct patients to the highest quality resources.

This study has several limitations. Our top-100 ranked list provides a snapshot of the websites returned by a subset of search engines on a particular day. Many search engines personalize results by location and other information about the user made available through the browser; our methodology attempts to minimize bias by eliminating this personalization. Thus, the top-100 list represents an example of the websites a patient may be likely found, in a relative order, but does not provide a definitive ranking and does not account for personal variation. Our study is also limited to websites in English, but we expect that the list of websites returned non-English speaking regions, and the associated quality of information, would vary. We also expect that multilingual populations may access information in multiple languages. Readability was assessed using text-based information, and did not assess supportive measures such as diagrams or glossaries, which may enhance content accessibility. Content completeness was scored across a range of topics of interest to patients; however, this may not always fairly reflect the purpose of sites that intentionally focus on a narrower scope of information or services, or whether those sites meet the needs of patients who have a similarly narrow scope of interest. A narrow site may receive a lower overall score even if the quality of information provided is very high and appropriate for the website’s purpose. For this reason, while the overall score provides one useful method of comparison for our purposes, it should not be considered a single definitive measure of quality or presented out of the context of its contributing metrics. Future work could more specifically evaluate the quality of resources for websites targeted at more specific topics like prevention and screening.

Lastly, top-100 list reflects 100 informational websites that patients are likely to encounter when conducting a thorough online search for cervical cancer information. To compile the top-100 list, any websites that were not intended to provide comprehensive patient information, such as news articles, social media content, and blogs, were excluded. However, patients may still look to such websites answers to their questions. In this study, the majority of websites linked to at least one social media platform on which they maintained a presence. This significant adoption of social-media platforms by websites suggests this may be an important mode of patient engagement. Our tool is currently limited to the evaluation of comprehensive informational style websites, and so social-media content and other forms of websites like blogs were not assessed. Few studies have examined the reliability of health information presented on social media, and to our knowledge, no other studies have assessed the quality of information about cervical cancer specifically in that domain. Thus, future work could seek to better understand the quality of information that patients might encounter on other kinds of platforms, such as social media or blogs, when seeking information about cervical cancer.

## Conclusions

This study presents a systematic evaluation of the quality of the top-100 returned websites related to cervical cancer in a search across Google, Yippee, and Dogpile. The use of basic design principles to support usability and interactivity is widespread use across most websites. However, support for informational accountability is often lacking, most notably with respect to information currency, use of citations, and disclosure of authorship. A majority of information is written at a high school reading level or above, which may impact accessibility for patients. Significantly, while some topics such as cervical cancer screening and prevention are well covered, several topics known to be important cervical cancer patients, such as prognosis and staging, are underrepresented. Most websites are accurate in the information they choose to present, but many lack accountability or recent updates. Notably, the most highly ranked websites returned by the search engines did not always contain the websites providing the most comprehensive information.

Our results highlight the strengths and limitations that health care providers should consider when directing patients to online resources for cervical cancer. These results can also help healthcare providers in educating patients to find and select high-quality resources when conducting searches on their own. For organizations that develop online cervical cancer resources, these results can be used to identify deficiencies and inform specific improvements to make information more accessible, comprehensive, and relevant to patients.
